# Accounting for population structure reveals ambiguity in the *Zaire Ebolavirus* reservoir dynamics

**DOI:** 10.1371/journal.pntd.0008117

**Published:** 2020-03-04

**Authors:** Bram Vrancken, Tony Wawina-Bokalanga, Bert Vanmechelen, Joan Martí-Carreras, Miles W. Carroll, Justus Nsio, Jimmy Kapetshi, Sheila Makiala-Mandanda, Jean-Jacques Muyembe-Tamfum, Guy Baele, Kurt Vermeire, Valentijn Vergote, Steve Ahuka-Mundeke, Piet Maes

**Affiliations:** 1 KU Leuven, Department of Microbiology, Immunology and Transplantation, Rega Institute for Medical Research, Division of Clinical and Epidemiological Virology, Leuven, Belgium; 2 Research and Development Institute, National Infection Service, Public Health England, Porton Down, Wiltshire, United Kingdom; 3 Ministère de la Santé, Kinshasa, Democratic Republic of the Congo; 4 Institut National de Recherche Biomédicale (INRB), Kinshasa, Democratic Republic of the Congo; 5 KU Leuven, Department of Microbiology, Immunology and Transplantation, Rega Institute for Medical Research, Laboratory of Virology and Chemotherapy, Leuven, Belgium; Institute for Disease Modeling, UNITED STATES

## Abstract

Ebolaviruses pose a substantial threat to wildlife populations and to public health in Africa. Evolutionary analyses of virus genome sequences can contribute significantly to elucidate the origin of new outbreaks, which can help guide surveillance efforts. The reconstructed between-outbreak evolutionary history of *Zaire ebolavirus* so far has been highly consistent. By removing the confounding impact of population growth bursts during local outbreaks on the free mixing assumption that underlies coalescent-based demographic reconstructions, we find—contrary to what previous results indicated—that the circulation dynamics of Ebola virus in its animal reservoir are highly uncertain. Our findings also accentuate the need for a more fine-grained picture of the Ebola virus diversity in its reservoir to reliably infer the reservoir origin of outbreak lineages. In addition, the recent appearance of slower-evolving variants is in line with latency as a survival mechanism and with bats as the natural reservoir host.

## Author summary

Because of its implications for awareness, surveillance and risk assessment of EBOV transmission to humans, the origin of emerging *Zaire ebolavirus* is investigated at each outbreak. To reliably do so requires a good understanding of the circulation dynamics of *Zaire ebolavirus* in its reservoir, which has yet to be determined. Here, we analyzed available full-length *Zaire ebolavirus* genomes from past and current outbreaks to infer the between-outbreak circulation dynamics while avoiding model misspecification by downsampling the data.

## Introduction

Ebola virus (EBV) is a filamentous, enveloped, and non-segmented negative-sense RNA virus that belongs to the genus *Ebolavirus* in the family *Filoviridae* with a genome length of 18.9 kb encoding seven proteins. This genus consists of five distinct species recognized by the International Committee on Taxonomy of Viruses (ICTV): *Zaire ebolavirus* (EBOV), *Sudan ebolavirus* (SUDV), *Bundibugyo ebolavirus* (BDBV), *Taï Forest ebolavirus* (TAFV), *Reston ebolavirus* (RESTV), with all but *Reston ebolavirus* causing disease in humans [1, 2]. Recently, a putative sixth species, *Bombali ebolavirus* (BOMV), has been discovered in free-tailed bats in Sierra Leone [3]. The disease caused by *Zaire ebolavirus* is called Ebola virus disease (EVD) and the EBOV strain circulates in sub-Saharan Africa with sporadic spillovers into human populations. The primary case of EVD can get infected from contact with the natural reservoir host and/or by hunting or consuming ebolavirus-infected bush meat [4, 5].

EVD is characterized by a severe and often lethal pathology. During the 2013–2016 EBOV outbreak in West Africa, the most extensive Ebola virus outbreak recorded to date, over 11,000 of more than 28,000 infected individuals succumbed to the disease [6]. Between 2014 and 2018, the Democratic Republic of the Congo (DRC or COD) has been struck three times with an EBOV outbreak: in July 2014 with 69 reported cases (49 deaths) near Boende town in the Equateur province, in May 2017 with 8 confirmed cases (4 deaths) in the Likati health zone, Bas-Uélé province, located in the north of the country and in May 2018 in the Equateur province with 54 confirmed cases (33 deaths). The currently ongoing outbreak declared on August 1st, 2018 in the North Kivu province is now the second largest EBOV outbreak on record with 3,309 confirmed cases and 2,130 deaths according to the report of February 11, 2020 from the DRCs’ Ministry of Health.

Evolutionary analysis of Ebola virus genome sequences remains of upmost importance to identify the putative origin of emerging EBOV. To reliably do so, requires a good understanding of the circulation dynamics of EBOV in its reservoir. Despite being discovered in 1976, the EBOV natural reservoir host is still not known with certainty, although a number of bat species have been identified as the most likely reservoir hosts [7–11]. Yet, much remains to be learned about the dynamics of its enzootic circulation [7, 12]. Previous studies have shown that EVD outbreak lineages share a common ancestor very close in time to its first detection in 1976 and that the virus has evolved rapidly over time [13, 14]. These inferences relied on coalescent models—a backwards-in-time process whereby lineages are merged going back in time as a function of the population size until only a single lineage remains [15]—to infer the past population dynamics from the genealogical relationships. Despite the wide use of coalescent models, only recently the possibly confounding effects of non-random sampling—which invalidates the free mixing assumption—were investigated [16–18] or model-wise accounted for [19–21]. For EBOV, for example, it has been shown that using a model that more adequately captures the tree-generative process by allowing for structure in the population can reduce estimation bias for the evolutionary rate [22].

Here, we analyzed EBOV full genomes, including a newly generated full-length genome sequence of the Likati EBOV outbreak (NCBI GenBank accession number MH481611) and those from the most recent outbreaks in the DRC (data have been deposited in GenBank under accession number MH733477 to MH733491, MK007329 to MK007344, and MH898466) to infer the between-outbreak circulation dynamics while avoiding model misspecification by downsampling the data.

## Materials and methods

### Ethics statement

The Ebola virus genome sequences used originated from other studies and were publicly available. One patient sample from the 2017 Likati outbreak was sequenced in this study. Unfortunately, the patient passed away a few days after sample collection. The data was analyzed anonymously.

### RNA extraction

RNA was extracted from a confirmed blood sample from the May 2017 Likati outbreak in DRC using the QIAamp Viral RNA mini kit (Qiagen Benelux, Antwerp, Belgium) following the manufacturer’s instructions with minor modifications. The sample was collected on 7 May 2017 and originated from a 22-year-old male (onset of disease 30 April 2017, deceased 8 May 2017). RNA was extracted from 50 μl whole blood diluted in 90 μl sterile water. Samples were inactivated in a cat. 3 glove box by adding 560 μl of Buffer AVL and 560 μl of 96% ethanol [23]. An extra washing step was performed by adding 500 μl of buffer AW2 to the spin column followed by centrifugation at 20,000 x g for 3 minutes before the RNA was eluted in 60 μl of buffer AVE.

### RT-PCR, amplicon purification and MinION sequencing

Two primer pools specific for EBOV and kindly provided by the ARTIC project (http://artic.network), were used to facilitate full genome sequencing by Oxford Nanopore Technologies (ONT) MinION. The Qiagen OneStep RT-PCR kit (Qiagen Benelux) was used with each of the EBOV-specific primer pools designed to generate and amplify overlapping amplicons, which cover the EBOV genome. Briefly, 15 μl of viral RNA template was added to a total reaction volume of 25 μl containing 5 μl 5X Qiagen OneStep RT-PCR buffer, 1 μl dNTP mix containing 10 mM of each dNTP, 1 μl Qiagen OneStep RT-PCR enzyme mix, 3 μl of one of the primer pools (0.015μM final concentration) and RNase-free water. The amplification profile involved a reverse transcription step at 45°C for 30 min, followed by PCR activation at 95°C for 15 min, 40 cycles of amplification (94°C, 10 sec; 65°C, 30 sec; 68°C, 4 min 30 sec) and a final extension of 10 min at 68°C. Pooled amplicons were cleaned-up with AMPure XP beads (New England Biolabs, Leiden, Netherlands), by washing 2 times with 70% ethanol and resuspended in 50 μl of RNase-free water. The purified DNA was quantified on a Qubit 1.0 fluorimeter (Thermo Fisher Scientific, Asse, Belgium) and libraries were prepared according to the ‘1D Genomic DNA by ligation (SQK-LSK108)’ kit and protocol supplied by ONT, Oxford, UK. MinION sequencing was performed with MinKNOW v2.0 (version 18.03.1) using R9.4.1 flow cells (ONT). After sequencing, reads were based-called with Albacore v3.0.1 and subsequently quality, tag and primer trimmed with Porechop v0.2.3. To construct the consensus sequence, a hybrid approach was used: *de novo* assembly was performed with Canu v1.7.0 and reference mapping was done with MiniMap2 v.2.16. Both assemblies were joined and Nanopolish v0.9.2 was used to refine the obtained draft consensus sequence. CLC Genomics Workbench v11.0 (Qiagen Benelux) was used to manually inspect and correct the obtained draft consensus sequence.

### Phylogenetic inference

**Fig 1:**
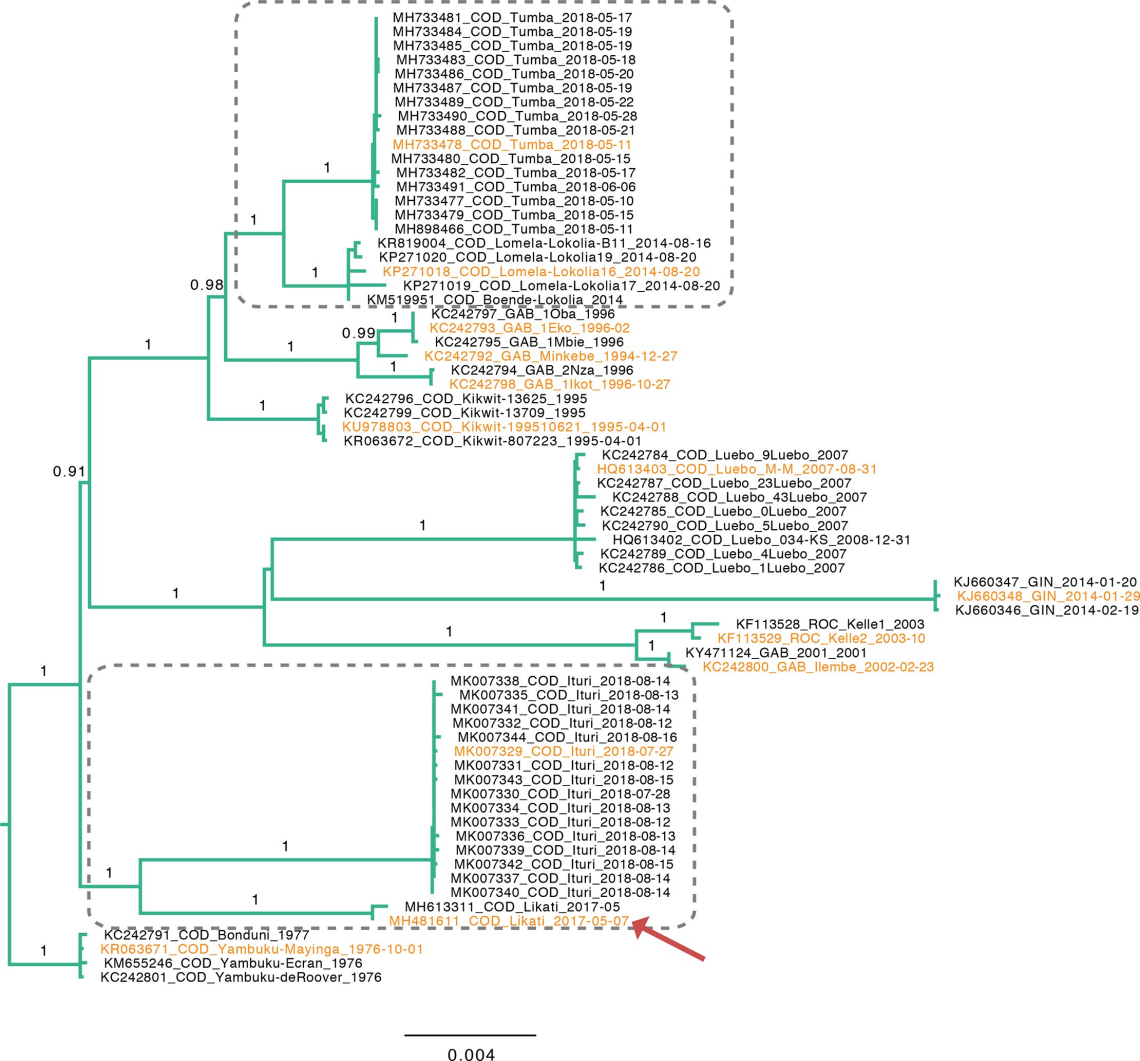
Evolutionary history of EBOV estimated from the available full genomes. The tree is rooted with the Yambuku outbreak sequences as outgroup [13, 14, 49]. Numbers above branches indicate support values obtained with the approximate likelihood ratio test. The red arrow highlights the position of the newly obtained full genome from the 2017 outbreak in Likati, COD. Representative sequences from each outbreak that were selected for further phylogenetic analyses are indicated in orange. Grey rectangles indicate lineages from outbreaks in the COD as of 2014 that appear to have been evolving at a slower pace than expected. The scale bar is in units of substitutions per site.

PhyML v3.0 [24] was used to infer an unrooted phylogenetic tree from the available EBOV near-complete genomes (> = 18654 nucleotides) using a general time-reversible (GTR) nucleotide substitution model [25] and a discrete Γ distribution [26] to capture among-site rate heterogeneity. Time-calibrated evolutionary histories were estimated using BEAST v1.10.4 [27]. A downsampled data set, from here on referred to as the EBOV2018 data set, was created by selecting one sequence per outbreak for which full genome data are available (Fig 1, orange highlight). This step is performed to avoid false positive test results when measuring the strength of the temporal signal, which can arise when closely related sequences are more likely to have been sampled at similar times [28, 29], as is the case for Ebola virus genomes sampled close together in time during an outbreak (Fig 1). This downsampling also prevents an impact of relatively brief periods of local virus population bursts during outbreaks among humans on the coalescent-based EBOV reservoir population size estimates [16, 22, 30].

As with previous analyses (e.g. [31, 32]), the alignment was divided into coding and non-coding partitions. The substitution process was modelled independently for the coding and non-coding partition according to the SDR06 [33] and HKY+Γ [26, 34] models, respectively. The generalized stepping stone marginal likelihood estimator (GSS MLE) as implemented in BEAST v1.10.4 [35] was used to compare the fit of an uncorrelated relaxed clock model with rates drawn from an underlying lognormal distribution [36] to that of a strict clock model. A CTMC Rate Reference prior was specified for the mean clock rate for both clock models [37]. Coalescent theory was used to infer the product of the effective population size N_*eff*_, which can be thought of as the relative genetic diversity [38], and the generation time τ; for clarity we refer to this product as the virus population size. As tests with the exponential growth model showed that the growth rate was indistinguishable from zero (the 95% highest posterior density, HPD, interval of the exponential growth rate encompassed zero), the population size was modelled using a constant population size model. The clock model and tree prior interact to determine the divergence dating [39], and it is possible that with small data sets the prior expectation on the population size is a non-negligible source of information. For this reason, we assessed the fit of more or less diffuse prior specifications (also with the GSS MLE), which were expressed as variations on the default proper prior on the constant population size hyperparameter (a lognormal distribution with mean 10 and standard deviation 100 in real space [40]). Specifically, diffuse lognormal distributions with (in real space) a mean of 10 or 100 and a standard deviation equal to the mean or equal to 10 times the mean were used. We provide an overview of the *a priori* expected credibility intervals for the population size with the various prior specifications in S1 Table.

## Results and discussion

### Slower evolution of particular EBOV lineages in the animal reservoir is in line with bats as reservoir hosts

Under the assumption that long-term EBOV evolution happens at a fairly constant rate [13, 14], it is expected that lineages from successive Ebola outbreaks are progressively more divergent from the EBOV most recent common ancestor. Hence, the noticeably small root-to-tip distances—relative to their sampling times—from the viruses sampled during EBOV outbreaks in the DRC since 2014 (Fig 1, grey boxes) challenge this assumption, and indicate that the 2017 and 2018 lineages, alike the 2014 Boende lineage [41], have been evolving in the reservoir at a slower rate than could be expected. Several observations indicate that the marked variation in EBOV evolutionary rates may be explained by the establishment of latent infections in its animal host. Firstly, latency has been observed in humans [42–44], where it is associated with a lower evolutionary rate [45]. Secondly, for Marburg virus, also a filovirus, it has been observed that outbreaks can be caused by variants that are genetically quasi identical to viruses detected in bats in previous years [46]. Hence, latency may be a survival strategy of filoviruses to overcome periods with reduced transmission opportunities, such as when host populations are smaller and contact rates lower [41]. Of note, as latency implies an animal host with a long life expectancy, this adds to the body of evidence [3, 7–9] pointing towards bats as the natural reservoir host of Ebola virus.

### The structured nature of EBOV outbreak sampling obfuscates between-outbreak reconstructions under the panmixis assumption

The effects of sampling strategy on phylogenetic reconstructions has been identified as an important challenge [47], and several works have investigated the potentially confounding effects of sampling biases when panmixis is assumed [16–19, 30]. A consistent outcome of these reports is that a temporal or geographic ascertainment bias can yield positively misleading results. Following their investigations into the impact of population structure on the temporal variation in the relative genetic diversity, Hall, Woolhouse (16) provide a set of guidelines, including the advice "*against the disproportionate inclusion of a large amount of sequence data from a single location*, *as this introduces false dynamics which should not be interpreted as a genuine decline in the size of viral populations*". This follows from the observation that an intense sampling of closely related sequences—such as during outbreaks—brings about a rapid succession of coalescent events just before the samples were obtained, which is reminiscent of a panmictic population that is declining in size. In turn, this causes an upwards bias in the evolutionary rate estimate and results in misleadingly recent tMRCAs [16]. Whereas Moller, du Plessis (22) show that allowing for population structure can account for this source of bias in a well-sampled within-outbreak setting, the limited number and poor temporal spacing of samples within outbreaks and concomitant lack of within-outbreak time signal make that a structured coalescent approach for now remains out of reach to infer the between-outbreak dynamics. Instead, we avoided an impact of relatively brief periods of local virus population bursts during outbreaks among humans on the coalescent-based EBOV reservoir population size estimates by selecting one representative sequence per outbreak (Fig 1).

To select the most appropriate model for inferring the between-outbreak evolutionary history from this data set, several combinations of clock models and prior expectations on the EBOV relative genetic diversity in its reservoir were investigated. An initial exploration revealed that only under the relaxed clock model and the population size prior with mean and st.dev. equal to 10 the between-outbreak mean clock rate estimates frequently spiked to values higher than the evolutionary rate estimate for the 2013–2016 outbreak [48] (S1 Fig). Such high estimates are unexpected given that the rate of EBOV evolution between outbreaks, which reflects long-term evolutionary processes, is generally anticipated to be lower than within outbreaks (see Holmes, Dudas (48) for details). Furthermore, uncertainty on the degree of among lineage rate variation under the latter model is high. This reflects in a bimodal coefficient of variation (CoV, the scaled variance in evolutionary rate among lineages [3]) that, on average, is almost twice as high as the CoV under the other models (.66 versus .35, .35 and .34). Together, this indicates that the rate of evolution cannot be reliably inferred under this model. Next, the relative fit was determined for all combinations of the three other population size prior specifications and a strict or relaxed clock model (S2 Table). In line with the observations from Fig 1, allowing for among branch rate heterogeneity decisively fits the data better than a strict clock model. There was also a differentiation in the fit of the prior expectations on the population size. The expectation with mean and st.dev. equal 100 provided the overall best fit. While there is strong support of this model over the prior with mean 10 and st.dev. 100 (ln(BF) = 3.01), the expectation with the same mean but a larger variance has a comparable fit (ln(BF) = 0.85). We report the results based on the best fitting model.

Comparison of the posterior evolutionary rate estimate, obtained from correct sampling times against the null distribution (obtained by randomly permuting the sampling dates), allows to determine whether or not a data set exhibits a significant time structure. In practice, this boils down to interpreting a continuous spectrum of possible extents of overlap between the null distribution and the tip-date informed posterior clock rate estimate. As a general criterion, there is significant time-structure when the 95% credible interval of the rate estimate obtained from correct sampling times does not overlap with that of the null distribution [28]. Such a date-randomization test shows that there is no overlap between the credible intervals, yet the tails of both posterior densities overlap (Table 1 and S2 Fig). This indicates that the EBOV2018 data possess a significant but not outspokenly strong temporal signal, which reflects in the wide range of plausible time to the most recent common ancestor (tMRCA) estimates (Table 1).

**Table 1:** Substitution rate and time to the most recent common ancestor (tMRCA) estimates. The substitution rate is expressed in substitutions/site/year. For the null estimate we averaged over all possible randomizations in a single analysis [52]. The mean and corresponding 95% highest posterior density (HPD) boundaries are given for each analysis.

substitution rate		tMRCA
correct sampling dates	randomized sampling dates	
1.40*10^−4^ (4.73*10^−5^–2.45*10^−4^)	2.16*10^−5^ (9.10*10^−6^–3.67*10^−5^)	1860 (1735–1967)

**Fig 2:**
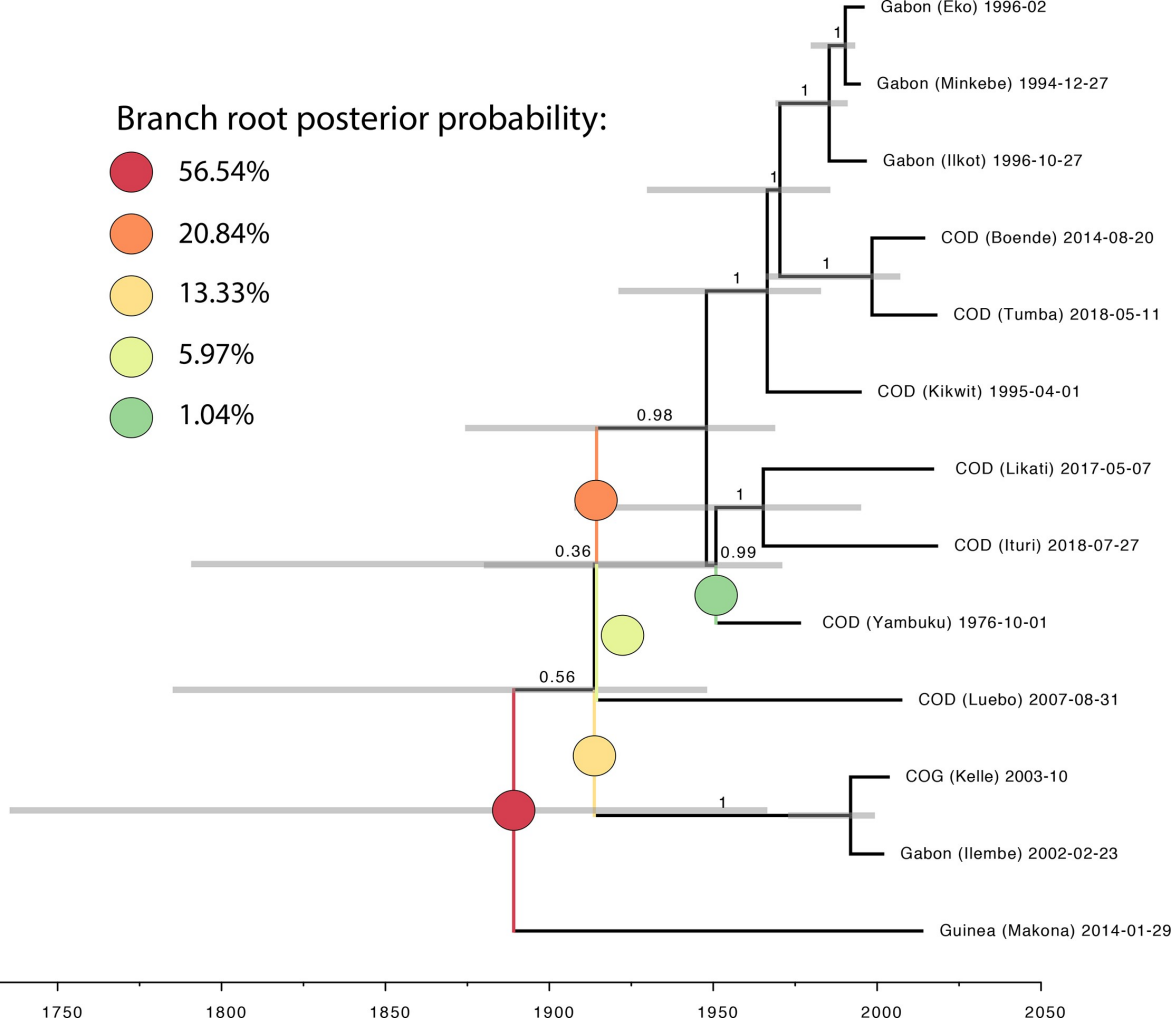
The between-outbreak epidemic history based on the EBOV2018 data set. Values next to branches represent their posterior probability. Bars show the 95% HPD interval for the internal node heights. Branch root posterior probabilities were obtained with RootAnnotator [49]. Branch root positions with >1% posterior probability are indicated in the phylogeny by colored circles and their support is given in the legend.

The inability to clearly inform the branch lengths comes with substantial uncertainty on the branch root position. Whereas the root previously was confidently placed on the branch to the Yambuku 1976 strain [13, 14, 49, 50], the support for this idea that all EBOV outbreaks are caused by descendants of a strain that much resembles the 1976 Yambuku variant is now ~1% (Fig 2). Rather, a topology that is compatible with the undetected endemic circulation of an EBOV lineage in West Africa, independent from strains from Central Africa [51] is the most plausible scenario. The second-best supported history is that the EBOV spillovers into the human population in the past decades are from strains of two clades that co-circulate in the EBOV reservoir.

To corroborate that the confounding between population structure and population size/evolutionary rate underlies the differences with previous results, we investigated whether these parameters are affected by the number of included samples per population (outbreak) as predicted when the populations indeed are highly structured. For this we used the best fitting evolutionary model for the EBOV2018 data set but now include at most 2, 3, 5 or all sequences per outbreak. As expected, we find an inverse relation between the maximal number of sequences per outbreak and the estimated population size (S3 Fig), and an increasing evolutionary rate with an increasing maximal number of sequences per outbreak (S4 Fig). In line with the observation by Baize, Pannetier (51), the between-outbreak topology varies with the evolutionary rate, and the topology becomes more consistent with that from earlier reports [13, 14, 49, 50] with the inclusion of increasing numbers of isolates per outbreak (S5 Fig).

**Fig 3:**
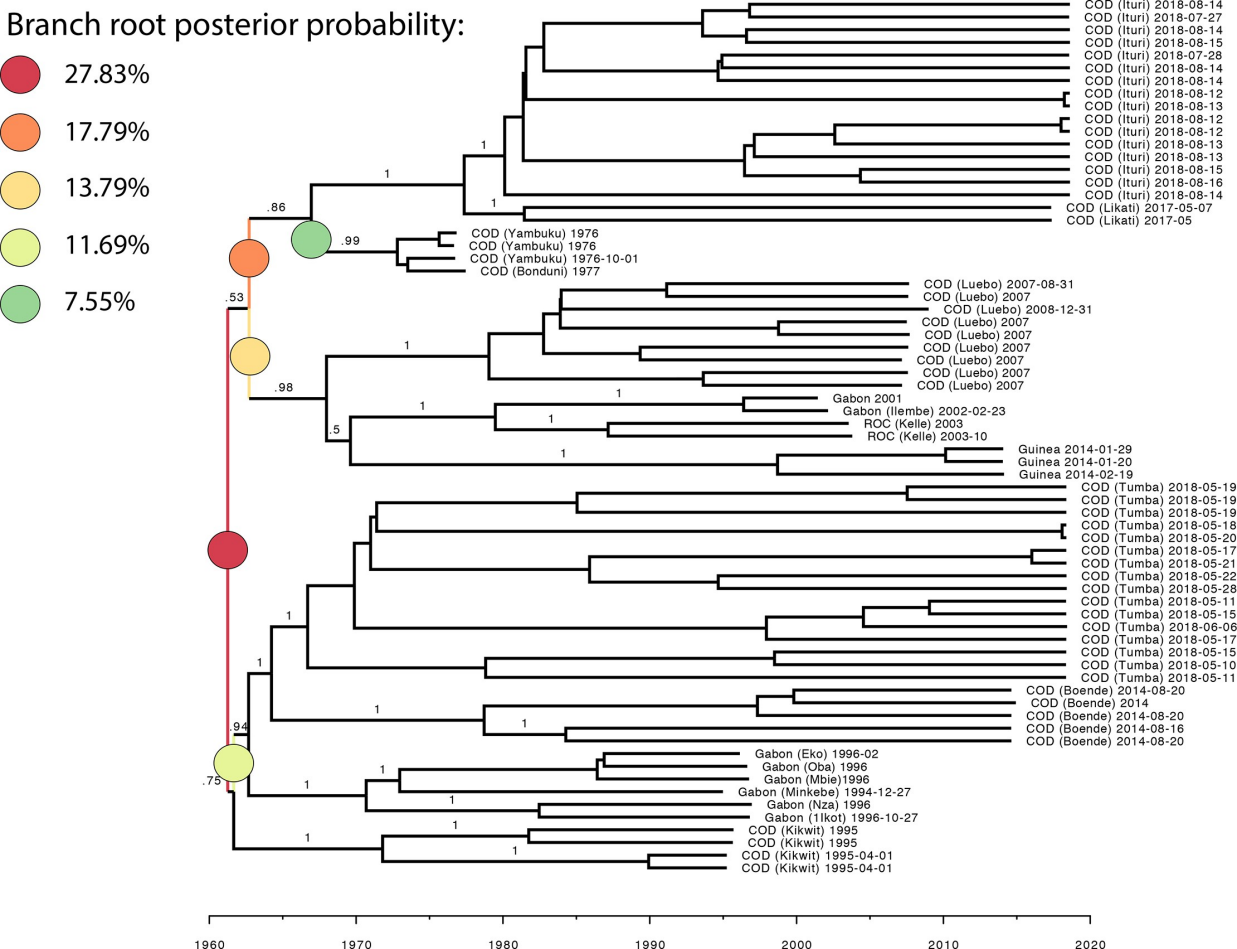
Time-scaled between-outbreak EBOV evolutionary history inferred without coalescent prior, represented by the maximum clade credibility summary phylogeny. Branch root posterior probabilities were obtained with RootAnnotator [49]. The 5 best supported branch root positions are indicated in the phylogeny by colored circles and their support is given in the legend. Numbers next to branches indicate their posterior support.

To further investigate whether the high confidence in a root on the branch to the 1976 outbreak follows from using a coalescent model that assumes panmixis, the complete data set was analysed with the same models as before except for the coalescent tree prior, which was replaced by an uniform prior that constrained the age of the tree to a 15 year interval between 1976 and 1961. If the coalescent model indeed drives the rooting in which the Yambuku lineage is an outgroup with respect to the other lineages, an uncertain rooting is expected in the absence of the coalescent model. The result from this analysis is summarized in Fig 3, and shows that the rooting indeed is highly uncertain under this condition.

There are indications that the frequently used uncorrelated relaxed clock models can be misleading when all branch rates are drawn from the same distribution, where in reality there may be multiple distributions with different means. A well-known example concerns the reconstruction of the influenza A virus gene flow among different host species, where allowing different host lineages to have independent rates of evolution is a prerequisite for reliable phylogenetic inference [21]. Latency may introduce such a rate variation effect, and there are indications that explicitly modelling the rate slow-down on the branches that likely experienced latency further increases the model fit and substantially reduces uncertainty on the divergence datings (http://beast.community/ebov_local_clocks.html, blog posted on May 17, 2019 nearly two months after the submission of our manuscript to this journal and about a year after the initial work was conducted). Allowing for latency in the clock model, however, does not impact the conclusions of this work.

## Conclusion

In summary, we highlight the need to appropriately account for the limits of coalescent models when reconstructing the EBOV between-outbreak evolutionary history, and that a broader sample of EBOV reservoir genetic diversity is required to reliably formulate hypotheses about the reservoir origins of outbreak variants. This can assist in identifying the factors that underlie the apparently recent emergence of slower-evolving, likely latent, EBOV variants.

Prevention and rapid control form the cornerstone of EBOV outbreak management. Yet, despite the potentially disastrous impact of an EBOV outbreak, vigilance for EBOV infection in West Africa may wane over time in favor for more incident pathogens that cause symptoms similar to those seen in EBOV infection (e.g. Lassa virus). This may be particularly true when it is thought that the previous outbreak resulted from a chance exportation event. By showing that the available virus genetic data are as yet unclear whether EBOV is endemic in West Africa or not, our results may help keep awareness at the highest possible level.

## Supporting information

S1 Table Expectations on the population size hyperparameter for the evaluated prior specifications. The mean and standard deviation of the lognormal distributions are given in real space. The values in the last column refer to the lower and upper bound of the corresponding confidence interval.
(DOCX)

S2 Table Model fit results. The relative fit of a strict clock model (SC) versus an uncorrelated relaxed clock model (UC) to the data was determined for several prior expectations on the population size. Higher values indicate a better model fit. The last column indicates the natural logarithm of the Bayes factor support in favor of a relaxed clock model. A ln(BF) difference >3 is generally considered as strong support in favor of a model [53]. The best fitting model is indicated in bold.
(DOCX)

S1 Fig Trace plots of the mean clock rate parameter estimated from the EBOV2018 data set. The horizontal red line corresponds to the 95% HPD of the West Africa outbreak rate estimate. The color-correspondence between the mean clock rate trace and the population size prior combination is in the legend. Spikes of the between-outbreak rate estimate above the within-outbreak rate estimate imply that unrealistically high mean clock rate values cannot be confidently rejected.
(PDF)

S2 Fig Quantifying the temporal signal. The correspondence between the posterior density and the use of correct or randomized sampling dates is as mentioned in the legend. The null estimate is shown in greyscale. The opaque sections correspond to the 95% credible intervals.
(PDF)

S3 Fig The inverse relation between the number of included sequences per outbreak and the estimated population size. (PDF)

S4 Fig The relation between the number of included sequences per outbreak and the evolutionary rate. (PDF)

S5 Fig Time-scaled between-outbreak EBOV evolutionary histories inferred using at most 2, 3, 5 or all available full genomes. The history is represented by the maximum clade credibility summary phylogeny. Branch root posterior probabilities were obtained with RootAnnotator [49]. The 4 best supported branch root positions with >1% posterior probability are indicated in the phylogeny by colored circles and their support is given in the legend.
(PDF)
