# Supplementary material for: Accounting for population structure reveals ambiguity in the Zaire Ebolavirus reservoir dynamics
Source: PLoS Negl Trop Dis. 2020 Mar 4;14(3):e0008117. doi: 10.1371/journal.pntd.0008117 (PMC7075637; doi:10.1371/journal.pntd.0008117)
Supplement: S1 Table — The mean and standard deviation of the lognormal distributions are given in real space. The values in the last column refer to the lower and upper bound of the corresponding confidence interval. (DOCX) [file pntd.0008117.s001.docx]

**Table S1: Expectations on the population size hyperparameter for the evaluated prior specifications.** The mean and standard deviation of the lognormal distributions are given in real space. The values in the last column refer to the lower and upper bound of the corresponding confidence interval.

| **mean** | **standard deviation** | **95% confidence interval** |
| --- | --- | --- |
| 10 | 10 | 1 - 36 |
|  | 100 | 0.02 - 67 |
| 100 | 100 | 14- 362 |
|  | 1000 | 0.1 - 670 |
