# Supplementary material for: Accounting for population structure reveals ambiguity in the Zaire Ebolavirus reservoir dynamics
Source: PLoS Negl Trop Dis. 2020 Mar 4;14(3):e0008117. doi: 10.1371/journal.pntd.0008117 (PMC7075637; doi:10.1371/journal.pntd.0008117)
Supplement: S2 Table — The relative fit of a strict clock model (SC) versus an uncorrelated relaxed clock model (UC) to the data was determined for several prior expectations on the population size. Higher values indicate a better model fit. The last column indicates the natural logarithm of the Bayes factor support in favor of a relaxed clock model. A ln(BF) difference >3 is generally considered as strong support in favor of a model [53]. The best fitting model is indicated in bold. (DOCX) [file pntd.0008117.s002.docx]

**Table S2: Model fit results.** The relative fit of a strict clock model (SC) versus an uncorrelated relaxed clock model (UC) to the data was determined for several prior expectations on the population size. Higher values indicate a better model fit. The last column indicates the natural logarithm of the Bayes factor support in favor of a relaxed clock model. A ln(BF) difference >3 is generally considered as strong support in favor of a model [53]. The best fitting model is indicated in bold.

| population size prior | SC | UC | ln(BF) |
| --- | --- | --- | --- |
| μ = 10, σ = 100 | -37173.32 | -37164.39 | 8.93 |
| μ = 100, σ = 100 | -37170.29 | **-37161.38** | 8.91 |
| μ = 100, σ = 1000 | -37171.20 | -37162.23 | 8.97 |
