# Supplementary figures and images for: Accounting for population structure reveals ambiguity in the Zaire Ebolavirus reservoir dynamics
Source: PLoS Negl Trop Dis. 2020 Mar 4;14(3):e0008117. doi: 10.1371/journal.pntd.0008117 (PMC7075637; doi:10.1371/journal.pntd.0008117)

# EBOV2018

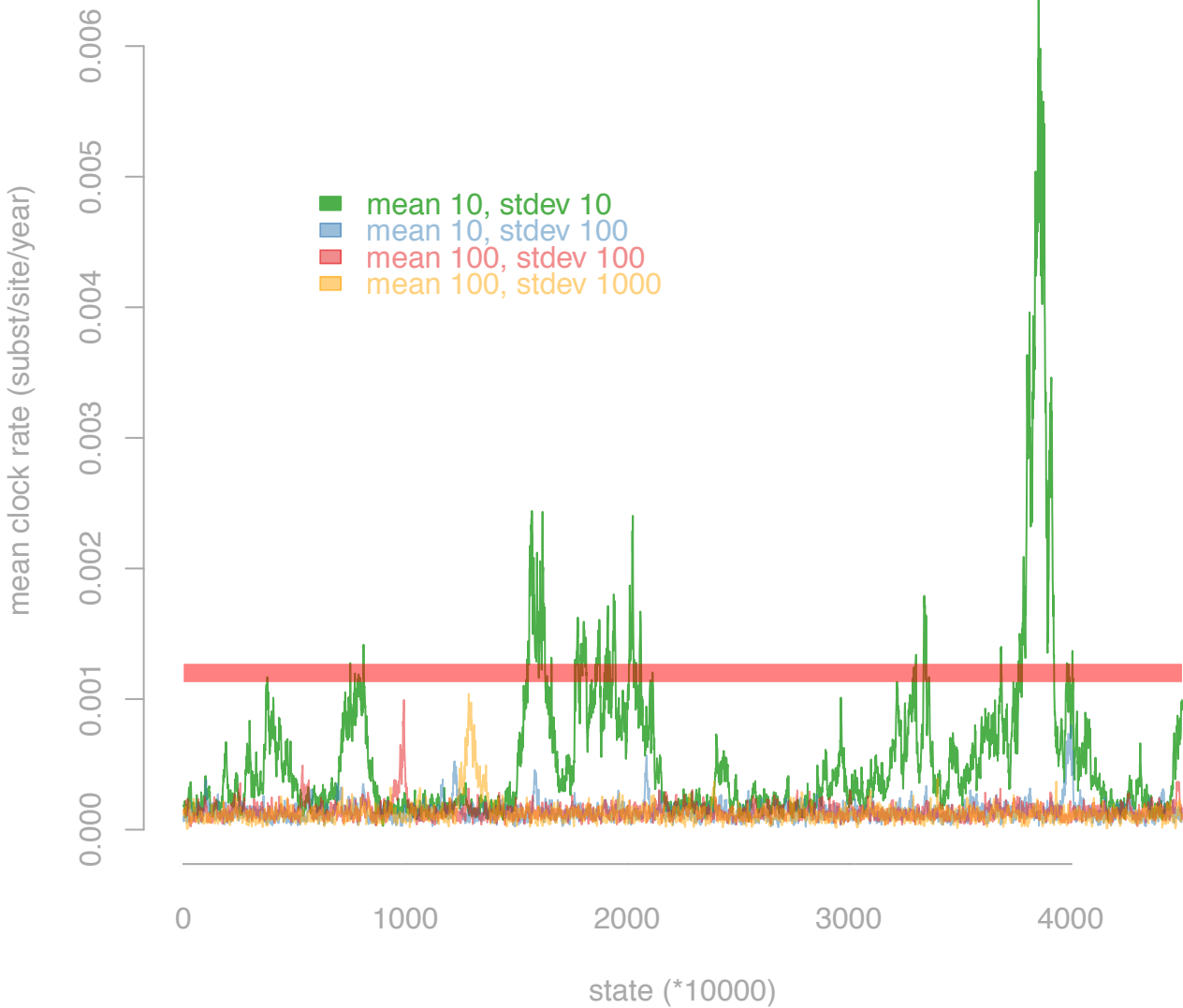

Supplement: S1 Fig — The horizontal red line corresponds to the 95% HPD of the West Africa outbreak rate estimate. The color-correspondence between the mean clock rate trace and the population size prior combination is in the legend. Spikes of the between-outbreak rate estimate above the within-outbreak rate estimate imply that unrealistically high mean clock rate values cannot be confidently rejected. (PDF) [file pntd.0008117.s003.pdf]

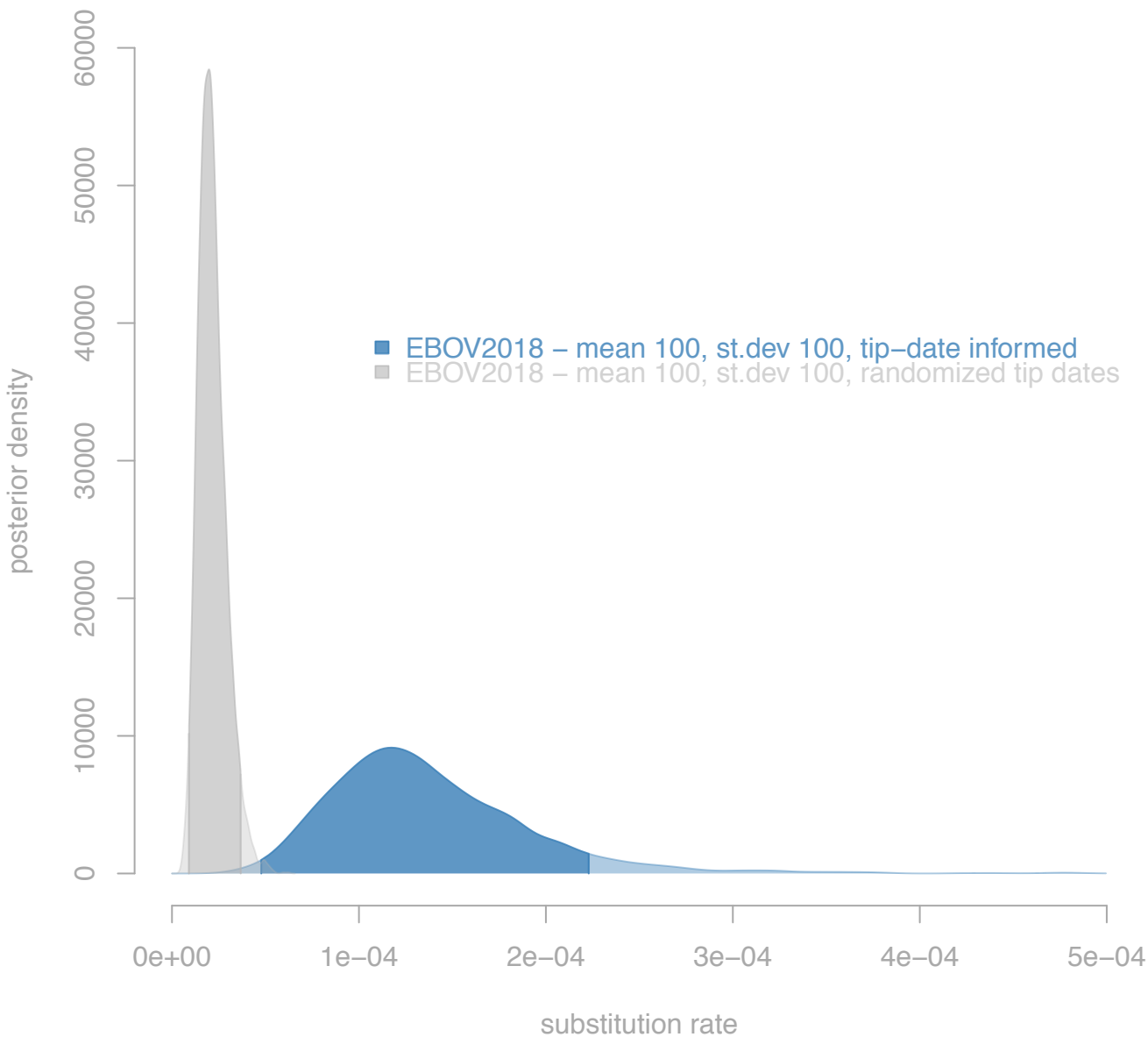

Supplement: S2 Fig — The correspondence between the posterior density and the use of correct or randomized sampling dates is as mentioned in the legend. The null estimate is shown in greyscale. The opaque sections correspond to the 95% credible intervals. (PDF) [file pntd.0008117.s004.pdf]

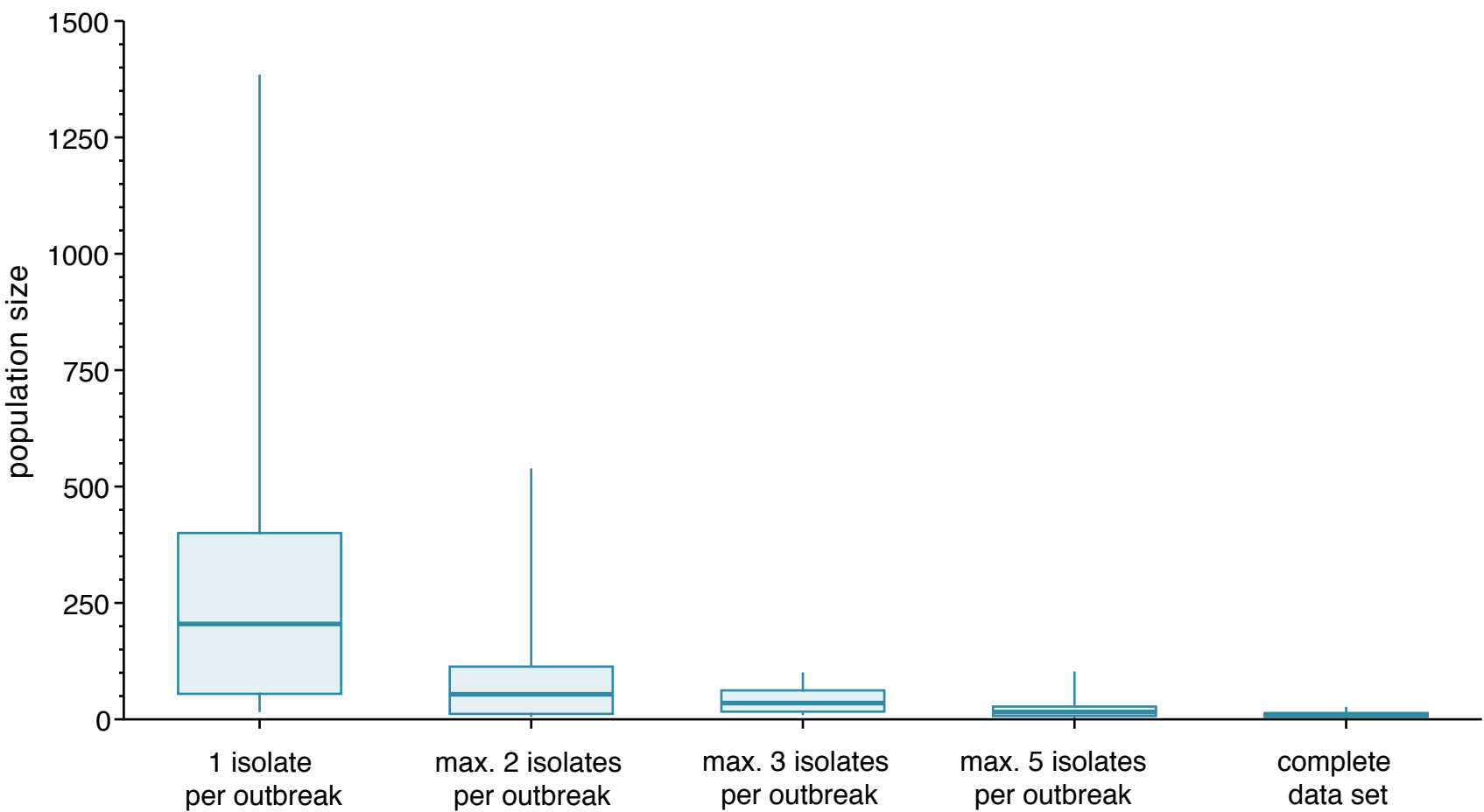

Supplement: S3 Fig — (PDF) [file pntd.0008117.s005.pdf]

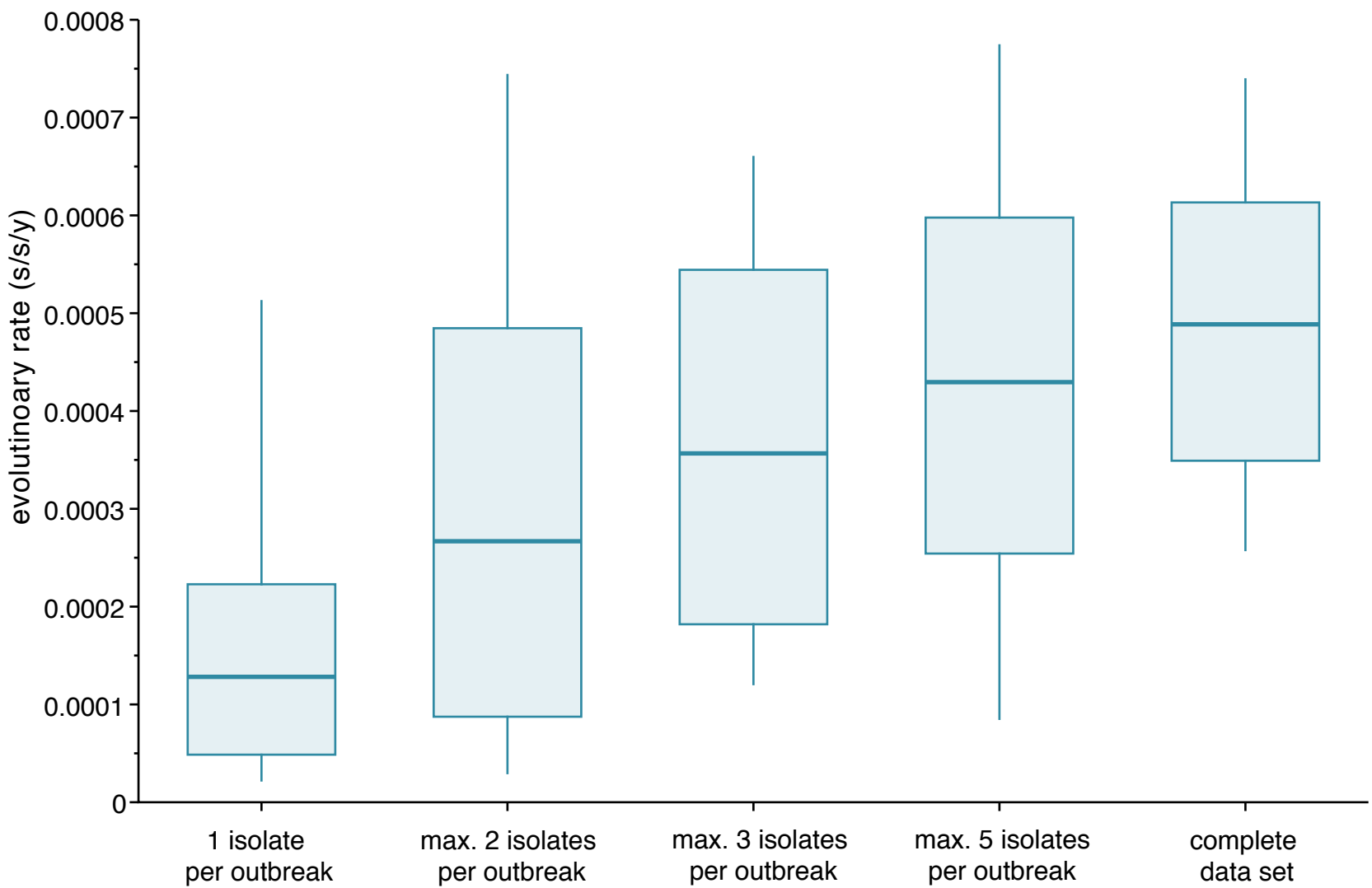

Supplement: S4 Fig — (PDF) [file pntd.0008117.s006.pdf]
