# Supplementary material for: Accounting for population structure reveals ambiguity in the Zaire Ebolavirus reservoir dynamics
Source: PLoS Negl Trop Dis. 2020 Mar 4;14(3):e0008117. doi: 10.1371/journal.pntd.0008117 (PMC7075637; doi:10.1371/journal.pntd.0008117)
Supplement: S5 Fig — The history is represented by the maximum clade credibility summary phylogeny. Branch root posterior probabilities were obtained with RootAnnotator [49]. The 4 best supported branch root positions with >1% posterior probability are indicated in the phylogeny by colored circles and their support is given in the legend. (PDF) [file pntd.0008117.s007.pdf]

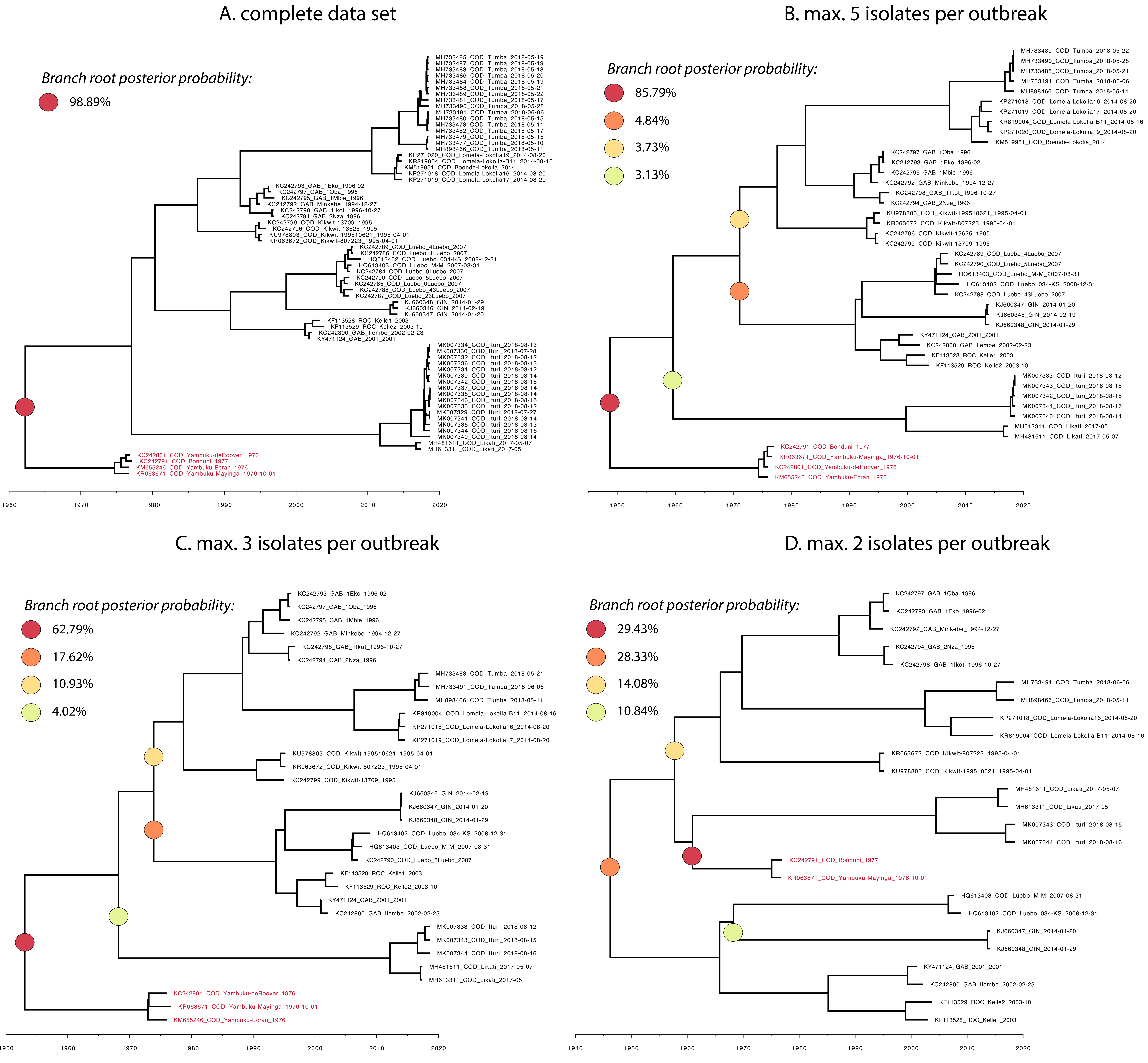

A. complete data set
Branch root posterior probability:
98.89%
MH733485_COD_Tumba_2018-05-19
MH733487_COD_Tumba_2018-05-19
MH733483_COD_Tumba_2018-05-18
MH733486_COD_Tumba_2018-05-20
MH733484_COD_Tumba_2018-05-19
MH733488_COD_Tumba_2018-05-21
MH733489_COD_Tumba_2018-05-22
MH733481_COD_Tumba_2018-05-17
MH733490_COD_Tumba_2018-05-28
MH733491_COD_Tumba_2018-06-06
MH733480_COD_Tumba_2018-05-15
MH733478_COD_Tumba_2018-05-11
MH733482_COD_Tumba_2018-05-17
MH733479_COD_Tumba_2018-05-15
MH733477_COD_Tumba_2018-05-10
MH898466_COD_Tumba_2018-05-11
KP271020_COD_Lomela-Lokolia19_2014-08-20
KR819004_COD_Lomela-Lokolia-B11_2014-08-16
KM519951_COD_Boende-Lokolia_2014
KP271018_COD_Lomela-Lokolia16_2014-08-20
KP271019_COD_Lomela-Lokolia17_2014-08-20
KC242793_GAB_1Eko_1996-02
KC242797_GAB_1Oba_1996
KC242795_GAB_1Mbie_1996
KC242792_GAB_Minkebe_1994-12-27
KC242798_GAB_1Ikot_1996-10-27
KC242794_GAB_2Nza_1996
KC242799_COD_Kikwit-13709_1995
KC242796_COD_Kikwit-13625_1995
KU978803_COD_Kikwit-199510621_1995-04-01
KR063672_COD_Kikwit-807223_1995-04-01
KC242789_COD_Luebo_4Luebo_2007
KC242786_COD_Luebo_1Luebo_2007
HQ613402_COD_Luebo_034-KS_2008-12-31
HQ613403_COD_Luebo_M-M_2007-08-31
KC242784_COD_Luebo_9Luebo_2007
KC242790_COD_Luebo_5Luebo_2007
KC242785_COD_Luebo_0Luebo_2007
KC242788_COD_Luebo_43Luebo_2007
KC242787_COD_Luebo_23Luebo_2007
KJ660348_GIN_2014-01-29
KJ660346_GIN_2014-02-19
KJ660347_GIN_2014-01-20
KF113528_ROC_Kelle1_2003
KF113529_ROC_Kelle2_2003-10
KC242800_GAB_Ilembe_2002-02-23
KY471124_GAB_2001_2001
MK007334_COD_Ituri_2018-08-13
MK007330_COD_Ituri_2018-07-28
MK007332_COD_Ituri_2018-08-12
MK007336_COD_Ituri_2018-08-13
MK007331_COD_Ituri_2018-08-12
MK007339_COD_Ituri_2018-08-14
MK007342_COD_Ituri_2018-08-15
MK007337_COD_Ituri_2018-08-14
MK007338_COD_Ituri_2018-08-14
MK007343_COD_Ituri_2018-08-15
MK007333_COD_Ituri_2018-08-12
MK007329_COD_Ituri_2018-07-27
MK007341_COD_Ituri_2018-08-14
MK007335_COD_Ituri_2018-08-13
MK007344_COD_Ituri_2018-08-16
MK007340_COD_Ituri_2018-08-14
MH481611_COD_Likati_2017-05-07
MH613311_COD_Likati_2017-05
KC242801_COD_Yambuku-deRoover_1976
KC242791_COD_Bonduni_1977
KM655246_COD_Yambuku-Ecran_1976
KR063671_COD_Yambuku-Mayinga_1976-10-01
1960
1970
1980
1990
2000
2010
2020
B. max. 5 isolates per outbreak
Branch root posterior probability:
85.79%
4.84%
3.73%
3.13%
MH733489_COD_Tumba_2018-05-22
MH733490_COD_Tumba_2018-05-28
MH733488_COD_Tumba_2018-05-21
MH733491_COD_Tumba_2018-06-06
MH898466_COD_Tumba_2018-05-11
KP271018_COD_Lomela-Lokolia16_2014-08-20
KP271019_COD_Lomela-Lokolia17_2014-08-20
KR819004_COD_Lomela-Lokolia-B11_2014-08-16
KP271020_COD_Lomela-Lokolia19_2014-08-20
KM519951_COD_Boende-Lokolia_2014
KC242797_GAB_1Oba_1996
KC242793_GAB_1Eko_1996-02
KC242795_GAB_1Mbie_1996
KC242792_GAB_Minkebe_1994-12-27
KC242798_GAB_1Ikot_1996-10-27
KC242794_GAB_2Nza_1996
KU978803_COD_Kikwit-199510621_1995-04-01
KR063672_COD_Kikwit-807223_1995-04-01
KC242796_COD_Kikwit-13625_1995
KC242799_COD_Kikwit-13709_1995
KC242789_COD_Luebo_4Luebo_2007
KC242790_COD_Luebo_5Luebo_2007
HQ613403_COD_Luebo_M-M_2007-08-31
HQ613402_COD_Luebo_034-KS_2008-12-31
KC242788_COD_Luebo_43Luebo_2007
KJ660347_GIN_2014-01-20
KJ660346_GIN_2014-02-19
KJ660348_GIN_2014-01-29
KY471124_GAB_2001_2001
KC242800_GAB_Ilembe_2002-02-23
KF113528_ROC_Kelle1_2003
KF113529_ROC_Kelle2_2003-10
MK007333_COD_Ituri_2018-08-12
MK007343_COD_Ituri_2018-08-15
MK007342_COD_Ituri_2018-08-15
MK007344_COD_Ituri_2018-08-16
MK007340_COD_Ituri_2018-08-14
MH613311_COD_Likati_2017-05
MH481611_COD_Likati_2017-05-07
KC242791_COD_Bonduni_1977
KR063671_COD_Yambuku-Mayinga_1976-10-01
KC242801_COD_Yambuku-deRoover_1976
KM655246_COD_Yambuku-Ecran_1976
1950
1960
1970
1980
1990
2000
2010
2020
C. max. 3 isolates per outbreak
Branch root posterior probability:
62.79%
17.62%
10.93%
4.02%
KC242793_GAB_1Eko_1996-02
KC242797_GAB_1Oba_1996
KC242795_GAB_1Mbie_1996
KC242792_GAB_Minkebe_1994-12-27
KC242798_GAB_1Ikot_1996-10-27
KC242794_GAB_2Nza_1996
MH733488_COD_Tumba_2018-05-21
MH733491_COD_Tumba_2018-06-06
MH898466_COD_Tumba_2018-05-11
KR819004_COD_Lomela-Lokolia-B11_2014-08-16
KP271018_COD_Lomela-Lokolia16_2014-08-20
KP271019_COD_Lomela-Lokolia17_2014-08-20
KU978803_COD_Kikwit-199510621_1995-04-01
KR063672_COD_Kikwit-807223_1995-04-01
KC242799_COD_Kikwit-13709_1995
KJ660346_GIN_2014-02-19
KJ660347_GIN_2014-01-20
KJ660348_GIN_2014-01-29
HQ613402_COD_Luebo_034-KS_2008-12-31
HQ613403_COD_Luebo_M-M_2007-08-31
KC242790_COD_Luebo_5Luebo_2007
KF113528_ROC_Kelle1_2003
KF113529_ROC_Kelle2_2003-10
KY471124_GAB_2001_2001
KC242800_GAB_Ilembe_2002-02-23
MK007333_COD_Ituri_2018-08-12
MK007343_COD_Ituri_2018-08-15
MK007344_COD_Ituri_2018-08-16
MH481611_COD_Likati_2017-05-07
MH613311_COD_Likati_2017-05
KC242801_COD_Yambuku-deRoover_1976
KR063671_COD_Yambuku-Mayinga_1976-10-01
KM655246_COD_Yambuku-Ecran_1976
1950
1960
1970
1980
1990
2000
2010
2020
D. max. 2 isolates per outbreak
Branch root posterior probability:
29.43%
28.33%
14.08%
10.84%
KC242797_GAB_1Oba_1996
KC242793_GAB_1Eko_1996-02
KC242792_GAB_Minkebe_1994-12-27
KC242794_GAB_2Nza_1996
KC242798_GAB_1Ikot_1996-10-27
MH733491_COD_Tumba_2018-06-06
MH898466_COD_Tumba_2018-05-11
KP271018_COD_Lomela-Lokolia16_2014-08-20
KR819004_COD_Lomela-Lokolia-B11_2014-08-16
KR063672_COD_Kikwit-807223_1995-04-01
KU978803_COD_Kikwit-199510621_1995-04-01
MH481611_COD_Likati_2017-05-07
MH613311_COD_Likati_2017-05
MK007343_COD_Ituri_2018-08-15
MK007344_COD_Ituri_2018-08-16
KC242791_COD_Bonduni_1977
KR063671_COD_Yambuku-Mayinga_1976-10-01
HQ613403_COD_Luebo_M-M_2007-08-31
HQ613402_COD_Luebo_034-KS_2008-12-31
KJ660347_GIN_2014-01-20
KJ660348_GIN_2014-01-29
KY471124_GAB_2001_2001
KC242800_GAB_Ilembe_2002-02-23
KF113529_ROC_Kelle2_2003-10
KF113528_ROC_Kelle1_2003
1940
1950
1960
1970
1980
1990
2000
2010
2020
